# The Role of Epoxyeicosatrienoic Acids in Diabetes Mellitus-Induced Impaired Vascular Relaxation of Aortic Rings in Ovariectomized Sprague-Dawley Rats

**DOI:** 10.1155/2019/5410108

**Published:** 2019-03-27

**Authors:** Dragan Manojlović, Ana Stupin, Anita Matić, Zrinka Mihaljević, Sanja Novak, Ines Drenjančević

**Affiliations:** ^1^Department of Surgery, General Hospital Vukovar, Vukovar, Croatia; ^2^Department of Physiology and Immunology, Faculty of Medicine, Josip Juraj Strossmayer University of Osijek, Osijek, Croatia; ^3^Department of Pathophysiology, Physiology, and Immunology, Faculty of Dental Medicine and Health, Josip Juraj Strossmayer University of Osijek, Osijek, Croatia

## Abstract

**Aim:**

The present study was aimed at determining if type 1 diabetes mellitus (DM) affects vascular function and at elucidating the mechanisms mediating vasorelaxation in both nonovariectomized and ovariectomized Sprague-Dawley (SD) rats.

**Materials and Methods:**

Eighty female SD rats were divided into four groups: nonovariectomized healthy (non-OVX-CTR) and diabetic (non-OVX-DM) rats and ovariectomized healthy (OVX-CTR) and diabetic (OVX-DM) rats. Bilateral ovariectomy was performed at the age of 5 weeks, and type 1 DM was induced by streptozotocin at the age of 6 weeks. At the age of 12 weeks, acetylcholine-induced relaxation (AChIR) was assessed in aortic rings in the absence/presence of L-NAME, Indomethacin, and MS-PPOH. Aortic tissue mRNA expression of eNOS, iNOS, COX-1, COX-2, thromboxane synthase 1 (TBXAS1), CYP4A1, CYP4A3, and CYP2J3, as well as plasma oxidative stress, was measured.

**Results:**

AChIR did not differ in non-OVX-DM rats compared to non-OVX-CTR ones. AChIR was significantly reduced in the OVX-DM group compared to the OVX-CTR group. MS-PPOH did not reduce AChIR in OVX-DM rats as it did in OVX-CTR ones. CYP4a3 mRNA expression in OVX-DM rats was significantly lower compared to that in the OVX-CTR group.

**Conclusions:**

Female sex hormones may protect vasorelaxation in type 1 diabetic rats. Type 1 diabetes impairs vasorelaxation in response to ACh in ovariectomized rats (but not in nonovariectomized rats) by affecting vasorelaxation pathways mediated by EETs.

## 1. Introduction

The most important complications of diabetes mellitus (DM) relate to vascular disease, which affects both microvasculature (neuropathy, diabetic nephropathy, and retinopathy) and macrovasculature (peripheral arterial disease, coronary artery disease) [1], while endothelial dysfunction is implicated in the pathogenesis of such diabetic vascular disorders [2, 3]. It has been shown that DM impairs vascular reactivity by increasing response to physiological vasoconstrictors and decreasing reactivity to vasodilators [4, 5]. Such vascular disturbances were demonstrated in different vascular beds and in response to various stimuli, e.g., impaired arteriolar response to acetylcholine (ACh) and to flow and increased contraction in response to noradrenalin (NA) in the aorta and skeletal and mesenteric arteries [6, 7]. Disturbed endothelium-dependent vasodilation in diabetic rats may occur due to compromised bioavailability of the normally protective vasodilator mediators, such as nitric oxide (NO). Besides NO, important vasodilators produced by the endothelium are metabolites of arachidonic acid (AA), such as cyclooxygenase-1- and cyclooxygenase-2- (COX-1- and COX-2-) derived vasodilator prostacyclin (PGI_2_), and in recent years much investigated cytochrome P450- (CYP450-) derived vasodilators, such as epoxyeicosatrienoic acids (EETs) [8]. Their biological roles in vascular function are extremely important—they serve as an endothelium-derived hyperpolarizing factor and have proangiogenic, anti-inflammatory, antiapoptotic, and profibrinolytic effects [9]. Contrasting data on the potential role of EETs in mediating vascular reactivity in various pathological states, including DM, have been published. Either EET-dependent vasodilation becomes an important compensatory mechanism in vessels with a lower bioavailability of NO (as they are in DM), or, in contrast, impaired vasodilation occurs due to decreased activity of EETs in animals with DM [10]. We have previously demonstrated that EETs have an important role in mediating restored relaxation responses of aortic rings in diabetic rats subjected to hyperbaric oxygen treatment [11, 12].

Premenopausal women are relatively protected against cardiovascular diseases (CVDs) compared to age-matched men, possibly because of higher circulating levels of female sex hormones (e.g., estrogen) which are believed to play a protective role by their favorable effect on vascular structure, function, and cell signaling [13–15]. However, it has been suggested that some of the estrogen effects on the endothelium may be modified or attenuated in various pathological states characterized by the increased level of oxidative stress [16, 17], which is also present in DM and has been shown to play a pivotal role in the development of diabetes complications [4–7]. It has still not been elucidated how the vasculoprotective role of estrogens is affected in women with DM who have a high incidence of CVDs. Moreover, there is a paucity of data on the effect of estrogens on the course and timeline of endothelial vascular changes during DM, which may ultimately result in clinically relevant micro- and macrovascular complications of DM.

Taking into account the possible effects of estrogen on the metabolic pathways important for vasorelaxation, the aims of the present study were (1) to determine if 6-week type 1 DM affects vascular function of rat aortas in both nonovariectomized (non-OVX) and ovariectomized (OVX) female Sprague-Dawley (SD) rats and (2) to elucidate at least some of the mechanisms mediating vascular relaxation in healthy and diabetic ovariectomized and nonovariectomized rats, with the emphasis on the role of EETs.

## 2. Materials and Methods

### 2.1. Experimental Animals

The animals were grown at the animal care facility of the Faculty of Medicine Osijek. All experimental procedures conformed to the “European Convention for the Protection of Vertebrate Animals Used for Experimental and Other Scientific Purposes” (Council of Europe number 123, Strasbourg 1985) and were approved by the Ethics Committee of the Faculty of Medicine Osijek.

A total of eighty female Sprague-Dawley (SD) rats were divided into four groups (twenty rats per group): (1) nonovariectomized controls (non-OVX-CTR), (2) nonovariectomized type 1 diabetic rats (non-OVX-DM), (3) ovariectomized controls (OVX-CTR), and (4) ovariectomized type 1 diabetic rats (OVX-DM). Animals were housed in a shoebox-style cages, had free access to standard rat chow and tap water, and were maintained on a 12 : 12 h light : dark cycle.

Bilateral ventral ovariectomized rats (OVX rats) were made at the age of 5 weeks according to the method described by Khajuria et al. [18]. Before surgery, the animals were anaesthetized with a combination of ketamine (75 mg/kg) and midazolam (0.5 mg/kg). The oviduct was tied with a silk suture (Syneture, SOFSILK 3-0, Tyco Healthcare Ltd., UK). The peritoneum and the muscle layers were sutured with an absorbable suture (Syneture, POLYSORB 3-0, Tyco Healthcare Ltd., UK), and the skin was sutured with one nonabsorbable suture (SOFSILK 3-0, Tyco Healthcare Ltd., UK). After surgery, the rats were placed individually in cages to recover for a period of one week. Metamizole sodium (1.5 mL/100 g i.m.) was used for postoperative analgesia.

Type 1 DM was induced by injecting streptozocin (60 mg/kg) intraperitoneally at the age of 6 weeks. Monitoring of the success of development of type 1 DM was done according to the protocol already described in our laboratory [12]. Only animals in which type 1 DM was successfully developed (glucose level higher than 33.3 mmol/L in most animals) were used in further experimental protocols.

### 2.2. Assessment of Acetylcholine-Induced Relaxation (AChIR) on Isolated Aortic Rings

At the age of 12 weeks, fifteen animals from each experimental group were used for functional experiments on isolated aortic rings. Prior to decapitation with a guillotine, rat weight was measured, and rats were anaesthetized with a combination of ketamine (75 mg/kg) and midazolam (2.5 mg/kg). The experiment was done according to the protocol already described in our laboratory [11, 12, 19]. Isolated aortic rings (4 rings of about 3-4 mm in length from each animal) were mounted in chambers containing Krebs-Henseleit solution warmed at 37°C and oxygenated with 95% O_2_/5% CO_2_ gas mixture. Passive tension for each ring was set at 2.0 g. After the initial test for vessel viability and endothelial integrity (precontraction with 10^−7^ M NA followed by relaxation with 10^−5^ M ACh), maximal contraction was induced with 60 mM KCl + 10^−7^ M NA. The ACh-induced relaxation (AChIR) protocol was performed on precontracted (10− 7 M NA) aortic rings by increasing the ACh concentration in the tissue bath (10^−9^ to 10^−5^ M ACh). The AChIR protocol was done in the absence and in the presence of one of the inhibitors: (1) the nitric oxide synthase (NOS) inhibitor, nitro-L-arginine methyl ester (L-NAME, 3 × 10^−4^ M); (2) the nonselective COX-1 and COX-2 inhibitor, Indomethacin (10^−5^ M); and (3) the selective EET epoxidation inhibitor, MS-PPOH (10^−5^ M), in the tissue bath. The relaxation was expressed as the percentage of the remaining contraction of the NA-induced vasoconstriction.

### 2.3. mRNA Expression of Enzymes Catalyzing Vasoactive Mediators in the Rat Aorta

Aortas of the remaining five rats from each group were used for the measurement of mRNA expression of enzymes catalyzing vasoactive mediators using quantitative real-time PCR (Bio-Rad CFX96) [19]. mRNA expression levels of eNOS and iNOS, COX-1, COX-2, thromboxane A synthase 1 (TBXAS1), CYP4A1, CYP4A3, and CYP2J3 were measured in all the four groups of female SD rats. Aorta samples were stored in RNAlater (Qiagene, Germany) at a temperature of -80°C until RNA isolation. RNA was isolated from a tissue homogenate by TRIzol Reagent (Life Technologies, USA) according to the protocol by Chomczynski and Sacchi [20]. RNA integrity was checked on 1% agarose gel, and concentration was measured with a NanoDrop 1000 spectrophotometer. cDNA was obtained with a High-Capacity Reverse Transcriptase kit (Applied Biosystems, USA) according to the protocol prescribed by the manufacturer. Gene expression was normalized to the expression of the housekeeping gene HPRT and further analyzed.

### 2.4. Oxidative Stress Measurements

Blood samples for oxidative stress measurement were collected immediately after decapitation, centrifuged at 3500 rpm, and stored at -80°C until use. For oxidative stress measurements, a spectrophotometric thiobarbituric acid-reactive substances (TBARS) method, which measures products of lipid peroxidation from serum samples, was used. Since the method is nonspecific because the other substances bind to TBA (including proteins), trichloroacetic acid (TCA) was added to the sample to precipitate the proteins, and after that the supernatant was used for the measurement [19, 21]. The absorbance of the sample was measured on NanoPhotometer P300 UV/VIS (Implen) at 572 and 532 nm with malondialdehyde (MDA) as a standard (*μ*M MDA).

### 2.5. Reagents

NA, ACh, L-NAME, and Indomethacin were purchased from Sigma-Aldrich. Ketamine and midazolam were obtained from Pfizer. Streptozocin was purchased from Sigma-Aldrich. The Krebs-Henseleit solution (composition: 113 mM NaCl, 4.7 mM KCl, 1.2 mM MgSO_4_ × 7H_2_O, 22 mM NaHCO3, 1.2 mM KH_2_PO_4_, 11 mM glucose, 2.5 mM CaCl_2_ × 2H_2_O, and 0.026 mM ethylenediaminetetraacetic acid (EDTA) (pH 7.4)) was prepared from EDTA and purchased from Sigma-Aldrich. CaCl_2_ × 2H_2_O and NaHCO_3_ were purchased from Merck KGaA, Darmstadt, Germany, with the rest of the chemicals purchased from Kemika, Zagreb, Croatia. MS-PPOH was a gift from John R. Falck, Southwestern Medical Center, Dallas, TX, USA. The chemicals used to determine the oxidative stress were thiobarbituric acid (TBA; Sigma-Aldrich, DE, USA), trichloroacetic acid (TCA; Panreac, Europe), and 1,1,3,3-tetramethoxypropane (TMP; Sigma-Aldrich).

### 2.6. Statistical Analysis

All data are summarized as means ± SEM. Two-way ANOVA and Bonferroni post hoc tests were used to test the differences in ACh-induced relaxation among the groups. Half maximal effective concentration (logEC50) ACh values were compared by Student *t*-tests or one-way ANOVA tests, when appropriate. One-way ANOVA and Tukey post hoc tests were used to test the difference in body weight, blood glucose, oxidative stress level, and gene expression among the groups. A probability of *P* ≤ 0.05 was considered to be statistically significant. SigmaPlot version 11.2 (Systat Software Inc., Chicago, IL, USA) was used for statistical analysis, and GraphPad Prism v5.0 (GraphPad Software Inc., La Jolla, CA, USA) was used for graphic presentation of the obtained results.

## 3. Results

### 3.1. Body Weight, Blood Glucose Level, and Oxidative Stress of Experimental Animals (at the Age of 12 Weeks)

Diabetic rats (both non-OVX and OVX) had smaller body weight compared to their corresponding controls. OVX-CTR rats had significantly higher body weight compared to non-OVX-CTR, while the body weight of OVX-DM rats was not significantly different from that of the non-OVX-DM rats (Table 1). As expected, the blood glucose level was significantly increased in both the non-OVX-DM and OVX-DM groups compared to their corresponding controls, which confirmed the successful development of the diabetes model in our experimental protocol. There was no difference in the blood glucose levels between the non-OVX-CTR and OVX-CTR or between the non-OVX-DM and OVX-DM groups of rats (Table 1). Ovariectomized rats, both OVX-CTR and OVX-DM, had increased TBARS compared to the corresponding non-OVX rats (Table 1). Also, TBARS was significantly increased in non-OVX-DM rats compared to non-OVX-CTR ones, while in OVX-DM rats, TBARS was not significantly different from that in OVX-CTR ones (Table 1).

### 3.2. Acetylcholine-Induced Relaxation (AChIR) of Isolated Rat Aortic Rings

**Figure 1:**
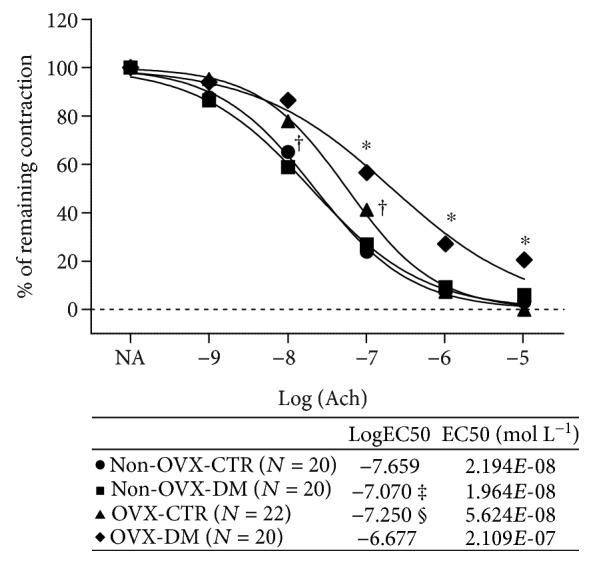
ACh-induced relaxation (AChIR) of isolated rat aorta rings in non-OVX-CTR and non-OVX-DM rats and OVX-CTR and OVX-DM rats. AChIR was significantly impaired in the OVX-DM group when compared to the non-OVX-DM group of rats at 10^−7^-10^−5^ M ACh concentration. Both non-OVX-DM and OVX-DM rats exhibited lower sensitivity to ACh compared to their corresponding controls (table within the panel). AChIR was significantly impaired in OVX-CTR compared to non-OVX-CTR (at 10^−7^-10^−5^ M ACh concentration) and OVX-DM compared to non-OVX-DM groups of rats (at 10^−7^-10^−5^ M ACh concentration). Both OVX-CTR and OVX-DM rats exhibited lower sensitivity to ACh compared to non-OVX groups, respectively (table within the panel). LogEC50 values (shown in the corresponding table) were compared by Student's *t*-test. ^∗^*P* < 0.05: OVX-CTR vs. OVX-DM, OVX-DM vs. non-OVX-DM. ^†^*P* < 0.05: OVX-CTR vs. non-OVX-CTR. ^‡^*P* < 0.05: non-OVX-CTR vs. non-OVX-DM, OVX-DM vs. non-OVX-DM. ^§^*P* < 0.05: OVX-CTR vs. OVX-DM, OVX-CTR vs. non-OVX-CTR. Ach concentration: 10^−9^ to 10^−5^ mol L^−1^. *N*: number of aortic rings. EC50 (mol L^−1^): half maximal effective concentration presents the concentration of ACh (mol L^−1^) which induces a response halfway between the baseline and maximum.

Figure 1 presents the baseline AChIR of isolated rat aortic rings in all the experimental groups of SD rats. While there was no significant difference in AChIR of isolated rat aortic rings between the non-OVX-CTR and non-OVX-DM groups of rats (Figure 1), the OVX-DM group of rats exhibited significantly reduced relaxation of isolated rat aortic rings in response to ACh compared to the OVX-CTR group (Figure 1). OVX-CTR rats exhibited reduced AChIR compared to non-OVX-CTR, and OVX-DM had reduced AChIR compared to non-OVX-DM rats (Figure 1). Furthermore, both non-OVX-DM and OVX-DM rats exhibited reduced sensitivity to ACh compared to their corresponding controls (table within Figure 1). Also, sensitivity to ACh was reduced in OVX-CTR rats compared to non-OVX controls, just as it was in OVX-DM rats compared to non-OVX-DM ones (table within Figure 1).

**Figure 2:**
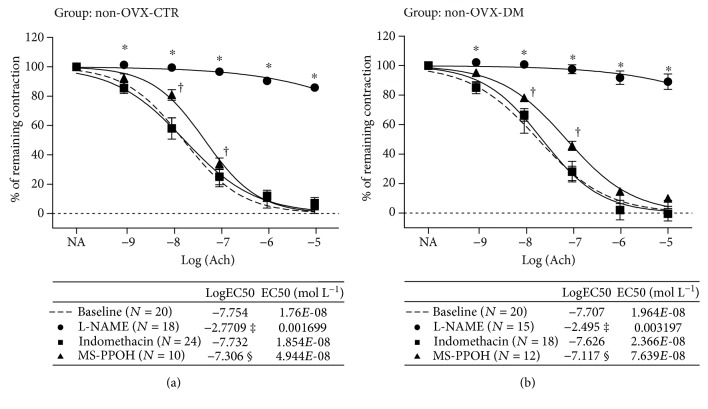
Mechanisms of acetylcholine-induced relaxation (AChIR) response of isolated rat aorta rings in non-OVX-CTR and non-OVX-DM rats. (a) and (b) present the relaxation of isolated aortic rings in response to ACh (presented as log [ACh] of ACh concentration (10^−9^ to 10^−5^ M)) in the non-OVX-CTR (a) and non-OVX-DM (b) groups of rats. The presence of L-NAME and MS-PPOH significantly reduced the AChIR of isolated rat aortic rings in the non-OVX-CTR group (a) and non-OVX-DM group (b) of rats. Indomethacin administration did not have any significant effects on AChIR in both the non-OVX-CTR (a) and non-OVX-DM (b) groups of rats. Data were compared by two-way ANOVA and Bonferroni post hoc tests. Sensitivity to ACh in the presence of L-NAME and MS-PPOH was significantly decreased compared to the basic response or response to ACh in the presence of Indomethacin in both the non-OVX-CTR and non-OVX-DM groups of rats. LogEC50 values were compared by one-way ANOVA followed by Holm-Sidak pairwise multiple comparison. Statistically significant (*P* < 0.05) AChIR in the presence of L-NAME (^∗^) or MS-PPOH (^†^) compared to the baseline ACh response. *P* < 0.05: ^‡^L-NAME vs. baseline, Indomethacin, and MS-PPOH; ^§^MS-PPOH vs. baseline and Indomethacin. Concentrations—ACh: 10^−9^ to 10^−5^ mmol L^−1^, L-NAME: 3 × 10^−4^ mmol L^−1^, Indomethacin: 10^−5^ mmol L^−1^, and MS-PPOH: 10^−5^ mmol L^−1^. *N*: number of aortic rings. EC50 (mol L^−1^): half maximal effective concentration presents the concentration of ACh (mol L^−1^) which induces a response halfway between the baseline and maximum.

**Figure 3:**
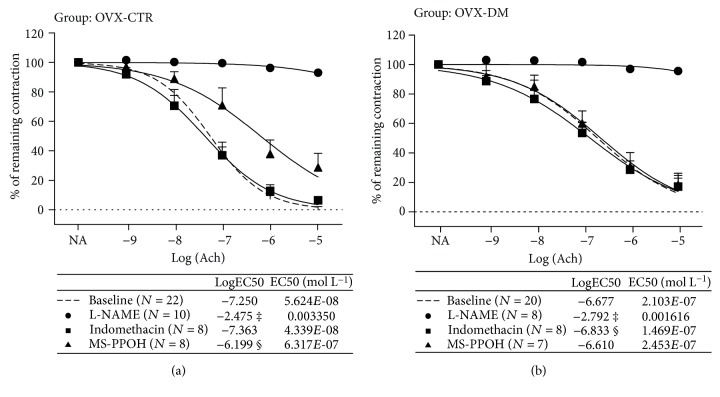
Mechanisms of the acetylcholine-induced relaxation (AChIR) response of isolated rat aorta rings in OVX-CTR and OVX-DM rats. (a) and (b) present the relaxation of isolated aortic rings in response to ACh (presented as log [ACh] of ACh concentration (10^−9^ to 10^−5^ M)) in the OVX-CTR (a) and OVX-DM (b) groups of rats. The presence of L-NAME and MS-PPOH significantly reduced the AChIR of isolated rat aortic rings in the OVX-CTR group (a), and only the presence of L-NAME significantly reduced the AChIR in the OVX-DM group (b) of rats. Indomethacin administration did not have any significant effects on AChIR in both the OVX-CTR (a) and OVX-DM (b) groups of rats. Data were compared by two-way ANOVA and Bonferroni post hoc tests. Sensitivity to ACh in the presence of L-NAME and MS-PPOH was significantly decreased compared to the basic response or response to ACh in the presence of Indomethacin in OVX-CTR, while in OVX-DM, sensitivity to ACh in the presence of L-NAME was significantly decreased compared to the basic response or response to ACh in the presence of Indomethacin or MS-PPOH. LogEC50 values were compared by one-way ANOVA followed by Holm-Sidak pairwise multiple comparison. Statistically significant (*P* < 0.05) AChIR in the presence of L-NAME (^∗^) or MS-PPOH (^†^) compared to the baseline ACh response. *P* < 0.05: ^‡^L-NAME vs. baseline, Indomethacin, and MS-PPOH; ^§^MS-PPOH vs. baseline and Indomethacin. Concentrations—ACh: 10^−9^ to 10^−5^ mmol L^−1^, L-NAME: 3 × 10^−4^ mmol L^−1^, Indomethacin: 10^−5^ mmol L^−1^, and MS-PPOH: 10^−5^ mmol L^−1^. *N*: number of aortic rings. EC50 (mol L^−1^): half maximal effective concentration presents the concentration of ACh (mol L^−1^) which induces a response halfway between the baseline and maximum.

The mechanisms mediating the AChIR response of isolated rat aortic rings in the experimental groups of rats are presented in Figures 2 and 3. In the non-OVX-CTR group of rats, the presence of L-NAME (eNOS inhibitor) and MS-PPOH (EET epoxidation inhibitor) significantly reduced AChIR, while Indomethacin administration did not have any significant effects on AChIR in the non-OVX-CTR group of rats (Figure 2(a)). The same results were obtained in the non-OVX-DM group of rats as well (Figure 2(b)). Sensitivity to ACh in the presence of L-NAME and MS-PPOH was significantly decreased compared to the basic response to ACh or response to ACh in the presence of Indomethacin, in both the non-OVX-CTR and non-OVX-DM groups of rats (tables within Figures 2(a) and 2(b)). In the OVX-CTR group of rats, the presence of L-NAME and MS-PPOH significantly reduced the AChIR of isolated rat aortic rings (Figure 3(a)), just as it did in non-OVX-CTR rats (Figure 2(a)), while Indomethacin administration did not have any significant effects on AChIR in the OVX-CTR group of rats (Figure 3(a)). On the other hand, in the OVX-DM group, the presence of L-NAME, but not MS-PPOH, significantly reduced the AChIR of isolated rat aortic rings (Figure 3(b)). Also, Indomethacin administration did not have any significant effects on AChIR in the OVX-DM group of rats (Figure 3(a)). Sensitivity to ACh in the presence of L-NAME and MS-PPOH was significantly decreased compared to the basic response or response to ACh in the presence of Indomethacin in both the OVX-CTR and OVX-DM groups of rats (in the OVX-CTR group of rats?) (table within Figure 3(a)), while in the OVX-DM group, sensitivity to ACh in the presence of L-NAME was significantly decreased compared to the basic response or response to ACh in the presence of Indomethacin or MS-PPOH (table within Figure 3(b)).

### 3.3. mRNA Expression of Enzymes Catalyzing Vasoactive Mediators in the Rat Aorta

mRNA expression of iNOS in rat aortic tissue was significantly increased in the non-OVX-DM group compared to the non-OVX-CTR group of rats (Table 2). COX-2 mRNA expression in thoracic aorta tissue was significantly decreased, and TBXAS1 mRNA expression significantly increased in OVX-DM rats compared to OVX-CTR ones (Table 2). CYP4A3 mRNA expression in thoracic aorta tissue was significantly decreased in OVX-DM rats compared to OVX-CTR ones (Table 2). eNOS, COX-1, CYP2j3, and CYP4A1 mRNA expression in thoracic aorta tissue did not differ between the non-OVX-CTR and non-OVX-DM, nor between the OVX-CTR and OVX-DM groups of rats (Table 2).

## 4. Discussion

DM is one of the major risk factors for the development and progression of CVDs [22]. Vascular alterations in both macro- and microcirculations are well-established complications in diabetic patients. Since endothelium dysfunction is considered to be a precursor and the earliest detectable outcome of CVDs [23], endothelium-dependent vasodilatation (also tested by the ACh-induced vascular relaxation of isolated rat aortic rings) is generally used as a parameter to assess endothelial function of arteries in different pathological states, including DM. Accordingly, endothelial dysfunction has been reported in both patients and experimental animals with diabetes [24–26]. On the other hand, studies investigating timeline and pathophysiological mechanisms involved in diabetes development (studies in animal models) brought conflicting results indicating normal or even enhanced endothelium-dependent responses [15, 27–29].

Salient finding of the present study is that 6-week type 1 DM did not affect the vascular reactivity of isolated aortic rings in female non-OVX SD rats, but it significantly impaired vasorelaxation in response to ACh in OVX rats. Even though type 1 DM in both non-OVX and OVX rats resulted in reduced sensitivity to ACh, impaired vasorelaxation in response to ACh was evident only in OVX-DM rats, but not in non-OVX-DM ones, compared to their controls. These results suggest that female sex hormones may have a protective effect (at least temporary) on vascular relaxation in type 1 DM rats. Furthermore, selective inhibition of EET epoxidation did not reduce AChIR in OVX-DM rats as it did in their corresponding OVX-CTR counterparts, indicating that type 1 DM per se impairs vasorelaxation in response to ACh in ovariectomized rats by decreasing the responsiveness to EETs. This conclusion is supported by the finding that type 1 DM downregulated CYP4A3 mRNA expression (specific CYP450 enzyme which catalyzes epoxygenation reactions of arachidonic acid) in thoracic aorta tissue in OVX-DM rats compared to their controls (OVX-CTR group). The results of this study confirmed the previous findings about estrogens' protective effect on vascular and endothelial function, since both OVX controls and type 1 diabetic rats exhibited reduced vasorelaxation in response to ACh compared to non-OVX-CTR and non-OVX-DM rats, respectively.

### 4.1. Interaction of Diabetes Mellitus and Estrogen

Lower incidence of CVDs in premenopausal women compared to age-matched men has been attributed to the protective effect of female sex per se and sex hormones (estrogens) on the cardiovascular system [13, 14]. Diabetic women have higher morbidity and mortality from CVDs compared to the general female population [30, 31]. On the other hand, recent large-scale clinical trials reported that postmenopausal women on hormone replacement therapy had significantly lower incidence of diabetes, despite no observed improvement in vascular outcomes [32, 33]. Evidently, DM and estrogen have important and complex interactions, but the potential mechanisms of these interactions are largely unknown.

The effects of nontreated type 1 DM and estrogen on body weight are well defined. Because insufficient insulin prevents the glucose uptake into the body's cells, one of the first symptoms of untreated type 1 DM is weight loss (besides polyuria, polydipsia, and polyphagia) [34], which was in accordance with the lower body weight in type 1 DM animals (both OVX and non-OVX) compared to their corresponding controls measured in the present study. Earlier studies reported that ovariectomy increased food intake, body weight, and body fat in experimental animals, and such estrogens' antiobesity effect was also observed in the present study results [35, 36]. Furthermore, vasculo- and endothelium-protective effect of estrogen has been well accepted, since a number of studies repeatedly demonstrated that ovariectomy in experimental animals impairs vascular relaxation in different vascular beds and in response to various stimuli [37, 38], which was confirmed with the present results that demonstrated that OVX rats (both CTR and DM) had impaired isolated aortic ring relaxation in response to ACh compared to non-OVX rats. In addition, our results have shown that both non-OVX and OVX type 1 diabetic rats had increased oxidative stress level (TBARS) compared to their healthy controls, which is in concordance with earlier studies reporting that DM is characterized by the increased production of reactive oxidative species (ROS). OVX rats (both CTR and DM) had significantly increased oxidative stress level compared to non-OVX rats, indicating that the presence of estrogen can act protectively against the adverse effect of oxidative stress (at least to a certain extent). In the present study, we have demonstrated that 6-week type 1 DM resulted in reduced sensitivity of aortic rings to ACh in both non-OVX and OVX rats. However, AChIR was not impaired in non-OVX-DM rats, only in OVX-DM ones compared to their corresponding controls, suggesting that estrogens may have a protective effect on vascular relaxation in type 1 diabetic rats. The question remains why increased oxidative stress level (due to type 1 DM) was not accompanied by impaired vascular reactivity in non-OVX rats, but was in OVX rats, or why vascular reactivity did not differ between non-OVX-CTR and OVX-CTR rats despite significantly different levels of oxidative stress. It was demonstrated that type 1 diabetes impaired vascular response to ACh in aortas of female rats (3 h exposure to high glucose, 1-week or 8-week DM duration) [39–41]. In contrast, others reported that the AChIR of aortic rings was not impaired in diabetic female mice (10-week type 1 DM duration) [42]. Furthermore, several studies demonstrated that aortic rings of OVX diabetic rats responded more weakly to ACh [15, 42], both in concordance with our findings. Besides their effects on NO production [15], estrogens affect COX pathways in the vascular wall, such as the PGI_2_-mediated pathway, as well as the activity of COX and prostacyclin synthase [43]. Moreover, it was reported that estrogen reintroduction partially improved vascular response, suggesting that estrogen or phytoestrogen could improve the endothelium-dependent vascular dilation in ovariectomized animals [15]. The beneficial effect of estrogen receptor agonists in specific aspects of vascular inflammation associated with type 1 DM was also reported [44].

### 4.2. Effects of Type 1 Diabetes on Mechanisms of AChIR in Female Nonovariectomized and Ovariectomized Rats

Although NO is a major mediator of endothelium-dependent vasodilation, changes in the NO-dependent pathway may not completely account for the development of endothelial dysfunction in DM [45]. In the present study, the NO-dependent part of vasorelaxation mechanisms was preserved and similarly represented in both type 1 diabetic groups, irrespective of ovariectomy. However, mRNA expression of iNOS in rat aortic tissue was significantly increased in the non-OVX-DM group compared to the non-OVX-CTR group of rats, which is in agreement with the increased iNOS production in the primary cultures of rat aortic smooth muscle cells (SMCs) isolated from type 1 diabetic rat aortas [44]. We speculate that the upregulation of iNOS and consequently increased NO synthesis may be a temporary compensatory mechanism dependent on the presence of estrogens, since AChIR was preserved in non-OVX type 1 diabetic rats (but not in OVX-DM rats). Furthermore, in the present study, there were no functional vascular differences in the part of the vasorelaxation mechanism that is COX-1- and COX-2-dependent or between healthy and type 1 diabetic female rats, irrespective of ovariectomy. However, COX-2 mRNA expression was significantly decreased, and TBXAS1 mRNA expression significantly increased in type 1 diabetic ovariectomized rats. It is known that TBXAS1 catalyzes the conversion of prostaglandin H2 to thromboxane A2, a potent vasoconstrictor [46]. Although the results of functional experiments in the present study do not support the role of COX-derived metabolites in vascular responses to ACh in ovariectomized type 1 diabetic rats, in the development of diabetic vascular damage, the effect of COX-dependent vasoconstrictors should not be neglected in long-term DM.

### 4.3. The Role of EETs in Vasorelaxation in Diabetic Rats

Some earlier studies suggested that DM interferes with endothelium-derived hyperpolarizing factor- (EDHF-) mediated vasodilation, but there is still a paucity of studies that differentiate the effect of DM on individual EDHFs (e.g., CYP450 metabolites of AA—EETs, potassium ion, H_2_O_2_, etc.) [10–12]. It has been reported that NO inhibits CYP activity, which may suggest that EET/EDRF pathways have a smaller role in a healthy blood vessel but become important vasodilators in vessels with a lower bioavailability of NO (such as in DM) [10]. Our group recently reported that EETs are an important but partial mediator of AChID of isolated aortic rings in male diabetic rats (6-week type 1 DM duration) [12]. Despite its potential compensatory vasodilator effect in conditions with decreased NO bioavailability, some research groups reported significantly decreased EET activity in animals with type 1 DM [10]. Furthermore, studies on animal models suggest that increased EET activity (by preventing their degradation or through dietary supplementation) [47] may attenuate DM-related disease progression through multiple mechanisms [10]. Hence, it seems that controlling the endogenous levels of EETs appears to be an effective strategy to protect the cardiovascular system against glucotoxicity [47]. A very recent study demonstrated that NO and EDHF, mainly EETs, dominate the ACh-induced vasodilation in renal arcuate arteries of lean Zucker male rats and that the contribution of both NO and EETs was impaired in diabetic rats [48]. In the present study, six weeks of type 1 DM induced downregulation of CYP4A3 isozyme, which specifically participates in the synthesis of EETs, in OVX rats, indicating that type 1 DM alters vascular reactivity in OVX female rats by inhibition of EET formation and showing the dependence of CYP450 4A3 activity on the glucose homeostasis. We have not found any studies investigating mechanisms of AChIR in isolated aortic rings or mRNA expression of enzymes catalyzing the synthesis of vasoactive mediators in aortic tissue in type 1 diabetic (non-OVX or OVX) female rats.

Estrogen modulates EDHF production and release (but not differentiating which one) [49]. Thus, according to available data, estrogen appears to exert protective effects on the nondiabetic endothelium by increasing the production or release of endothelium-derived relaxing factor (NO, EDHF, and PGI_2_) and decreasing the release or actions of endothelium-derived contracting factors (PGF_2_*α*, TXA_2_, and ANG II) [49, 50]. However, it should be considered that not all arteries respond the same way to estrogen, and since estrogen affects so many endothelial processes, it is imperative to gain a better understanding of the molecular mechanisms by which estrogen modulates vascular responses (in different vascular beds) in both health and disease states, including DM.

In conclusion, this is the first study (1) to show that 6 weeks of type 1 DM impaired aortic ring reactivity to ACh only in OVX, but not in non-OVX female SD rats, suggesting that female sex hormones may have a protective effect (at least temporary) on vascular relaxation in type 1 DM rats; (2) to measure significantly decreased CYP4a3 mRNA expression in thoracic aorta tissue of OVX-DM rats compared to their controls (OVX-CTR group); and (3) to demonstrate that the selective EET epoxidation inhibitor did not reduce AChIR in OVX-DM rats as it did in their OVX controls, together indicating that 6-week type 1 diabetes duration in OVX rats reduces EET synthesis and thus impairs the vasorelaxation of the isolated rat aortic ring in response to ACh in OVX rats precisely by reducing its responsiveness to ETTs. Our results represent a step to more extensive knowledge and understanding of the role of female sex hormones and regulatory properties of EETs in the macrovascular function of healthy and type 1 diabetic female rats.

## Figures and Tables

**Table 1:** Body weight, blood glucose, and oxidative stress level of experimental animals (at the age of 12 weeks).

Parameters	Non-OVX-CTR	Non-OVX-DM	OVX-CTR	OVX-DM
Body weight, g	240 ± 6^†^	183 ± 13^∗^	336 ± 5	208 ± 19^∗^^†^
Blood glucose, mmol/L	6.6 ± 0.2	30.8 ± 1.7^∗^	6.3 ± 0.1	30.7 ± 1.4^∗^
TBARS, *μ*mol MDA	0.4 ± 0.2^†^	1.1 ± 0.1^∗^	1.7 ± 0.1	2.0 ± 0.2^†^

Data are presented as mean ± SEM. OVX: ovariectomized; CTR: control; DM: diabetes mellitus; TBARS: thiobarbituric acid-reactive substances; MDA: malondialdehyde. ^∗^*P* < 0.05: CTR vs. DM; ^†^*P* < 0.05: non-OVX vs. OVX.

**Table 2:** Relative mRNA expression of eNOS, iNOS, COX-1, COX-2, TBXAS1, CYP2J3, CYP4A1, and CYP4A3 in thoracic aorta tissue.

Parameters	Non-OVX-CTR	Non-OVX-DM	OVX-CTR	OVX-DM
eNOS	0.096 ± 0.014	0.150 ± 0.034	0.137 ± 0.019	0.122 ± 0.013
iNOS	0.001 ± 0.3*E* − 3	0.026 ± 0.018^∗^	0.003 ± 0.002	0.004 ± 0.001
COX-1	0.134 ± 0.013	0.128 ± 0.041	0.094 ± 0.028	0.100 ± 0.007
COX-2	0.159 ± 0.062	0.124 ± 0.080	0.135 ± 0.073	0.624 ± 0.169^†^
TBXAS1	1.115 ± 0.152	1.173 ± 0.111	1.288 ± 0.135	1.555 ± 0.118^†^
CYP2J3	2.641 ± 0.297	1.932 ± 0.427	1.86 ± 0.178	1.949 ± 0.164
CYP4A1	0.0617 ± 0.017	0.053 ± 0.021	0.057 ± 0.020	0.029 ± 0.004
CYP4A3	1.4*E* − 6 ± 2.5*E* − 7	0.8*E* − 6 ± 2.8*E* − 7	2.9*E* − 6 ± 4.2*E* − 7	1.2*E* − 6 ± 7.7*E* − 7^†^

Data are presented as mean ± SEM. OVX: ovariectomized; CTR: control; DM: diabetes mellitus; eNOS: endothelial nitric oxide synthase; iNOS: inducible nitric oxide synthase; COX-1: cyclooxygenase-1; COX-2: cyclooxygenase-2; TBXAS1: thromboxane A synthase 1; CYP2J3: cytochrome P450 2J3; CYP4A1: cytochrome P450 4A1; CYP4A3: cytochrome P450 4A3. ^∗^*P* < 0.05: non-OVX-CTR vs. non-OVX-DM. ^†^*P* < 0.05: OVX-CTR vs. OVX-DM.

## Data Availability

The experimental data in the numerical form (presented as graphs and tables) used to support the findings of this study are included within the article.
